# *Healthcare* for Older Adults, Where Are We Moving towards?

**DOI:** 10.3390/ijerph18126219

**Published:** 2021-06-08

**Authors:** Francisco José Tarazona-Santabalbina, Sebastià Josep Santaeugènia Gonzàlez, José Augusto García Navarro, Jose Viña

**Affiliations:** 1 Departmanet of Geriatric Medicine, Hospital Universitario de la Ribera, Carretera de Corbera km 1, 46600 Alzira, Spain; 2 Centro de Investigación Biomédica en Red Fragilidad y Envejecimiento Saludable (CIBERFES), 28029 Madrid, Spain; jose.vina@uv.es; 3 Chronic Care Program, Health Department, Generalitat de Catalunya, 08002 Barcelona, Spain; sebastia.santaeugenia@gencat.cat; 4 Central Catalonia Chronicity Research Group (C3RG), Centre for Health and Social Care Research (CESS), Universitat de Vic–University of Vic-Central University of Catalonia (UVIC-UCC), C. Miquel Martí i Pol, 1, 08500 Vic, Spain; 5 Consorci de Salut i Social de Catalunya, 08022 Barcelona, Spain; jagarcia@segg.es; 6 Spanish Society of Geriatrics and Gerontology, 28006 Madrid, Spain; 7 Freshage Research Group, Department of Physiology, Faculty of Medicine, Institute of Health Research-INCLIVA, University of Valencia, 46010 Valencia, Spain

Since the end of World War II, science has not stopped progressing. Health and social advances have increased human longevity as never before [1]. We live longer, partially due to an increase in our knowledge of the physiological processes of successful aging, but also due to the pathophysiological processes of unhealthy aging. Among the least favorable aging trajectories, a syndrome that stands out from the gerontological and geriatric perspective is frailty. Since Linda Fried described the physiological cycle of this geriatric syndrome in 2001 [2], the presence of frailty in the elderly has been associated with a loss of functionality, hospital admissions, disability, mortality and institutionalization in nursing homes. In fact, it is a more robust predictor of adverse events than some variables that are still used abnormally in clinical inertia, such as chronological age [3]. This ageism remains in force in clinical practice, as has been possible to verify with the COVID-19 pandemic [4] that has devastated the planet. As it has been published, the elderly found it difficult to access the necessary clinical resources due to a simple matter of age.

Our Special Issue deals with the following issue: how to improve healthcare for our elders. One of the reviews included could not be more revealing in this regard, detailing the epigenetic factors linked to successful trajectories of aging [5]. Our life expectancy depends mainly on environmental factors, but we should not forget how the environment modifies our genetic expression through epigenetic mechanisms. Identifying the molecular and cellular epigenetic mechanisms can contribute to the early detection of changes or deficits associated with the different aging trajectories, including those that lead to frailty. The era of biomarkers has arrived. The biological clock shows the states of robustness and frailty in adults. With this information, clinicians can predict adverse events and improve decision-making processes.

Frailty, as should be remembered, is associated with multiple geriatric syndromes, including polypharmacy and pharmacological iatrogenesis. The periodic review of the pharmacological history of the elderly should be part of usual clinical routine. In this context, evaluating and reducing the anticholinergic load of prescribed medications has a positive effect on the cognitive status of our older adults and also reduces the incidence of some adverse events, such as respiratory infections [6]. Likewise, the analysis of frailty allows access to key prognostic information, in both community-dwelling and hospitalized older adults [7]. Similarly, advances in geriatric knowledge allow us to predict walking recovery after hip fracture surgery [8]. Thus, variables such as age and the estimation of anesthetic risk (due to its link with the prevalence of comorbidity), independence in walking prior to the fracture, the presence of cognitive impairment and pressure ulcers (both geriatric syndromes), the delay in surgery and early mobilization (two of the great challenges of clinical management), and the destination at discharge strongly influence gait recovery. Falls is another important geriatric syndrome and is linked to hip fracture incidence. Even if undervalued by the clinical practice, falling has important social and health implications, among which are the non-negligible associated costs. Falls are considered normal in the elderly by a great number of clinicians; this ageist principle is gradually being broken as indicated by the significant number of articles published between 2010 and 2020 on the matter [9]. These published studies should highlight the implication of geriatric factors in falling, such as sarcopenia, cognitive status, frailty, depression and fear of falling. The prevention of falls in older adults continues to be a great public health challenge.Accustomed to associating frailty with functional and clinical factors, clinicians often forget the social conditioning factors and constructs, such as social frailty [10]. Social frailty is associated with a worse clinical, nutritional, psycho-affective, cognitive situation, and with lower life satisfaction. In fact, physical activity is linked with social and psychological factors, such as the presence of depressive symptoms [11], the perception of fatigue, loneliness and social isolation. In this Special Issue, two studies pay attention to clinical application of thermal points. In older adults with dementia and verbal fluency impairments, thermal sensation could be useful to communicate with patients [12]. Moreover, thermotherapy could be effective in pain control [13].

Finally, interdisciplinary teams develop an important role in complex processes management, such as elective colorectal cancer surgery [14]. Holistic approaches, with a correct continuity of care, prolongs the benefits of enhanced recovery after surgery (ERAS) protocols, in the nutritional state and in hemoglobin blood levels, reducing transfusion rates.

This Special Issue offers different manuscripts to readers trying to improve life satisfaction, quality of life and life expectancy in older adults in different scenarios. It is up to us to achieve these goals. We are sure that these interesting papers will contribute to improve the clinical practice. Enjoy these articles.

## Data Availability

Not applicable.
